# Limits of Peripheral Blood Mononuclear Cells for Gene Expression-Based Biomarkers in Juvenile Idiopathic Arthritis

**DOI:** 10.1038/srep29477

**Published:** 2016-07-07

**Authors:** Laiping Wong, Kaiyu Jiang, Yanmin Chen, Teresa Hennon, Lucy Holmes, Carol A. Wallace, James N. Jarvis

**Affiliations:** 1 Department of Pediatrics, University at Buffalo, Buffalo, NY, USA; 2 Department of Pediatrics, University of Washington, Seattle, WA, USA; 3 Genetics, Genomics, & Bioinformatics Program, University at Buffalo, Buffalo, NY, USA

## Abstract

Juvenile Idiopathic Arthritis (JIA) is one of the most common chronic disease conditions affecting children in the USA. As with many rheumatic diseases, there is growing interest in using genomic technologies to develop biomarkers for either diagnosis or to guide treatment (“personalized medicine”). Here, we explore the use of gene expression patterns in peripheral blood mononuclear cells (PBMC) as a first step approach to developing such biomarkers. Although PBMC carry many theoretical advantages for translational research, we have found that sample heterogeneity makes RNASeq on PBMC unsuitable as a first-step method for screening biomarker candidates in JIA. RNASeq studies of homogeneous cell populations are more likely to be useful and informative.

The importance of developing biomarkers to assist in the diagnosis or to monitor the efficacy of therapy of adults and children with rheumatic diseases has been long recognized. Thus, the emergence of technologies to study gene expression on a genome-wide scale held considerable promise that, by surveying patterns of gene expression in an unbiased way, novel biomarkers might be developed and/or therapies individualized to maximize efficacy^1^,^2^,^3^. In adult rheumatic diseases, there has been some success in using this approach, for example, in predicting patients with rheumatoid arthritis who will or will not respond to therapy directed against TNF alpha^4^,^5^. Our group has also demonstrated the feasibility of being able to broadly predict treatment response at 6 months in children with new-onset polyarticular JIA based on patterns of whole blood gene expression at the time of clinical presentation^6^. Despite these advances, no useful biomarkers have come into general clinical use in pediatric rheumatology from data generated using gene expression profiling with hybridization-based microarrays.

In the past 5 years, there has been a growing trend toward using RNASeq as the preferred method for transcriptional analysis, even for biomarker development^7^. RNASeq carries several advantages over microarrays, providing a broader dynamic range and more comprehensive survey of the transcriptome^8^, including disease-associated splice variants^9^,^10^ and non-coding RNA species. We have recently reported the ability to properly classify patients with JIA as to disease status (active disease versus remission) from RNASeq data from peripheral blood neutrophils with as few as 3 samples of each phenotype^11^, something that we found more challenging using conventional microarrays^12^,^13^. However, the question arises whether similar accuracy can be obtained using PBMC, which are somewhat easier to prepare and which are sometimes considered more germane to the JIA disease process than neutrophils. However, PBMC present problems of their own in RNASeq studies. Whereas transcriptome profiling of neutrophils allows one to work with a relatively homogeneous cell population, PBMC represents a broad spectrum of cell types that may vary in numbers between individual patients. While projects like ENCODE and Roadmap Epigenomics have shown us that there are broad commonalities in the transcriptomes of these different cell types, there are also distinct differences that form the basis of differences in cellular function^14^,^15^. Thus, when comparing two phenotypes, if gene *x* is shows higher expression in T cells, but much lower expression B cells in one of the phenotypes compared to the other, then significant differences in expression may not be detected by either count based or fragment length based methods for analyzing RNASeq data^16^,^17^,^18^. Thus, in addition to the inter-patient variability that challenges biomarker development in JIA^19^, PBMC may add another level of variability that interdicts their use for this purpose. To address these issues, we performed deep RNA sequencing on PBMC of JIA patients to test the feasibility of using RNASeq as a first step to identify candidate biomarkers for diagnosis or treatment stage in polyarticular JIA^20^. We used two different sequencing facilities and independent patient cohorts in order to address generalizability and reproducibility issues, both critical for biomarker development.

## Results

We used samples from three independent cohorts (A, B and C) for this study. In cohort A, we studied 8 samples each for children: i) with newly diagnosed active untreated disease (ADU); ii) with active disease who had been on treatment (ADT); iii) who fit criteria for clinical remission on medication (CRM); and 8 healthy controls (HC). For the cohort B, we studied 9 patients with active disease on treatment and 10 patients in clinical remission on medication, each child of cohort B was studied on 2 occasions (blood was taken on 2 time points, denoted as CRM_B1 and CRM_B2). In the cohort C, there were 8 samples in each of the three different JIA states and 8 HC subjects.

Children designated as having active disease all had synovitis, as indicated by the presence of warmth and synovial thickening, in at least one joint. We used the Wallace criteria^20^ to determine CRM. That is, the CRM state was defined as inactive disease (no evidence of synovial, uveitis, or laboratory abnormalities that could be attributed to active JIA) that had been maintained for at least 6 continuous months.

### Transcriptomic comparison between treated and remission patients

We first sought to determine whether we could identify biomarker candidates that might unambiguously identify patients whose disease remained active while they were on treatment (ADT) from those who had achieved remission. Having such biomarkers would be useful in guiding decisions about how long to continue therapy and when it might safely be discontinued. In order to identify transcriptome changes that occur after treated patients achieve the remission (CRM) stage, we attempted to identify differently expressed genes (DEG) by comparing ADT and CRM. Criteria for remission are described in the Methods section.

**Figure 1:**
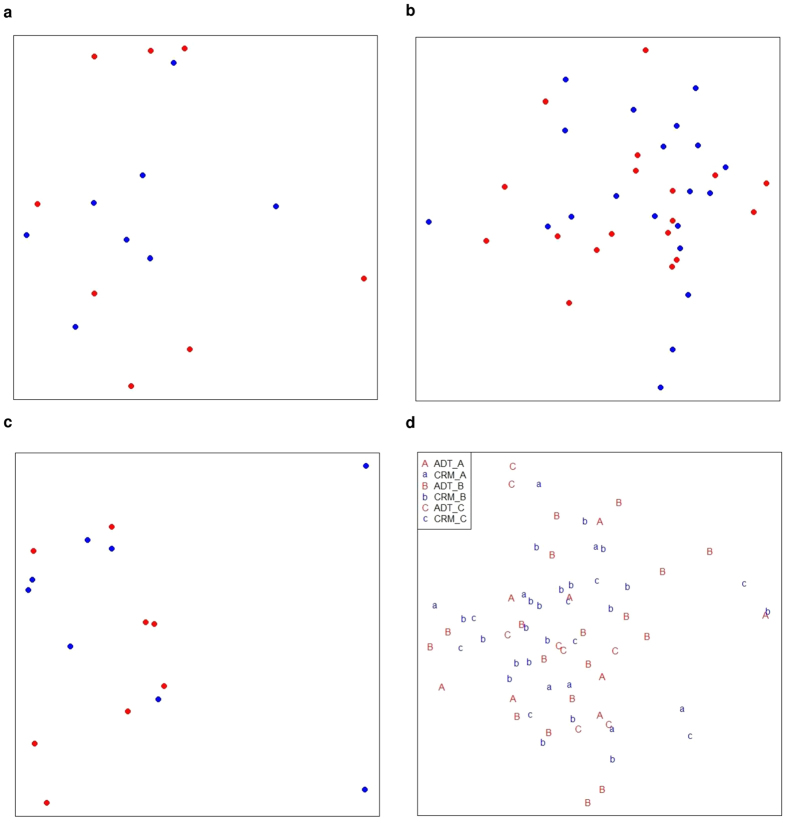
Multi dimensional scaling (MDS) analysis for the comparison of ADT and CRM. MDS plot of ADT versus CRM for samples from (**a**) cohort A, (**b**) cohort B and (**c**) cohort C and (**d**) merging of three cohorts with batch effect removal. Red represents ADT samples and blue indicates CRM individuals. Euclidean distance was used to measure between samples dissimilarities over gene expression values. ADT and CRM samples are randomly scattered with no distinguishable cluster formed for three cohorts as well as the combined sample set of three cohorts.

Prior to DEG analysis, we previewed the distributions of the ADT and CRM samples by constructing multi-dimensional scaling (MDS) plots over gene expressions. The two groups of samples scattered randomly without forming separable clusters (Fig. 1) when we analysed the three cohorts. This preview indicates that it is hard to identify DEG that unambiguously differentiate the ADT and CRM states. Nevertheless, we tried to identify potential DEG by using voom, running the analysis on each cohort independently. Very few DEGs were identified in any of the three cohorts using multiple different computational methods (Supplementary Table 1).

**Figure 2:**
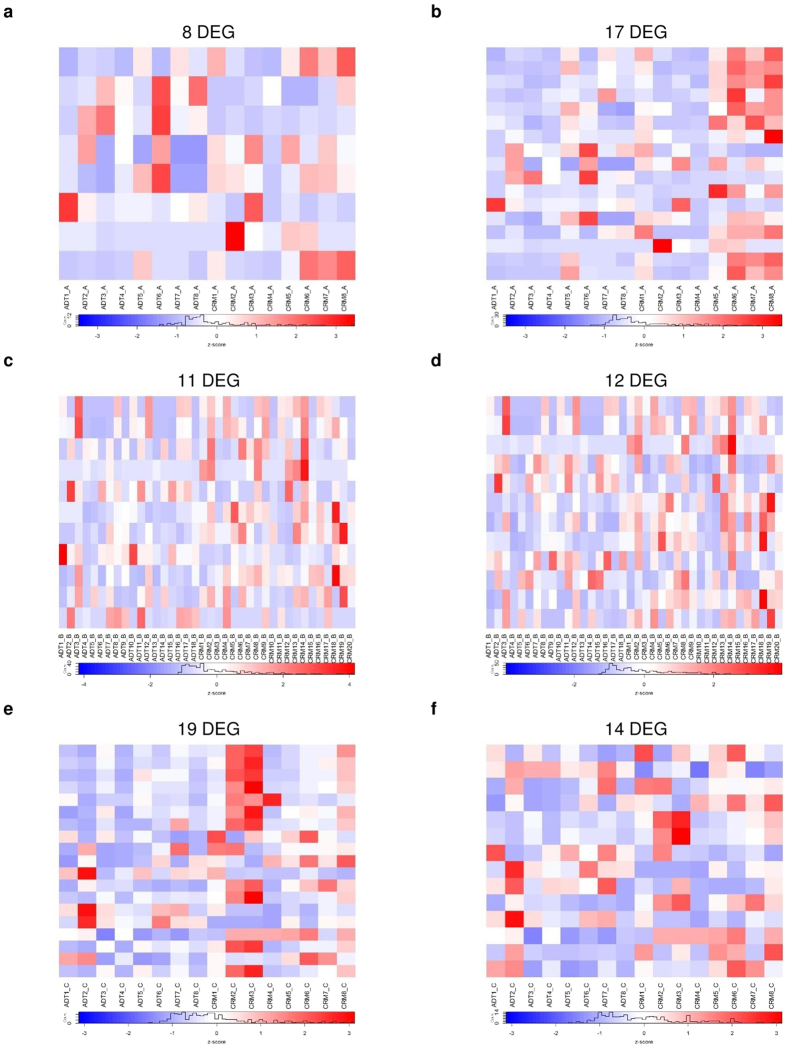
Heatmap plot of differentially expressed genes (DEG) detected by voom for the comparison of ADT and CRM samples. Heatmap of DEG discovered by voom only for (**a**) cohort A, (**c**) cohort B, (**e**) cohort C. Heatmaps of DEG discovered by voom with sample quality weighting for (**b**) cohort A, (**d**) cohort B and (**f**) cohort C. Title of each heatmap indicates number of DEG defined by p-value ≤ 0.05 and fold change ≥2 cutoff. Sample quality weighting did not improve DEG discovery.

We next adopted the sample weighting method recently reported by Liu *et al.*^21^ to reduce inter sample variation prior to DEG identification to improve DEG analysis. This approach provided no improvement in DEG discovery (Supplementary Table 1), as inter-sample variations remained problematic, as shown in Fig. 2. Adding to these obstacles were issues of reproducibility, as, we found no common DEG among the three cohorts. This finding was also likely due to variability between ADT and CRM subjects in terms their transcriptomes and not technical replication failure in the DEG search.

There are multiple computational approaches that can be used to identify DEG from RNASeq data^22^,^23^,^24^. We therefore used other DEG discovery tools to rule out the possibility that voom was incapable of dealing with data sets as variable as PBMC RNASeq. We chose another four DEG analysis methods that included cuffdiff2^25^, DESeq2^26^, EBSeq^27^ and edgeR^28^ (Supplementary Table 2), and each approach yielded few DEG in the ADT versus CRM comparison (Table 1). Furthermore, as with the voom analysis, we observed low overlapping rates between paired DEG detection methods and no common DEG across all methods (Supplementary Table 1).

We next attempted to combine samples across the three cohorts, on the assumption that larger sample sizes might better enable DEG identification. In this analysis, we used voom with sample quality weighting and applied batch effect removal before calling DEG. Once again, we were unable to identify DEG that might serve as useful biomarkers of remission (CRM), given the inter sample variation. Four DEGs were identified with adjusted p-values of ≤0.3 and fold change (FC) ranging between 1.4 and 1.6. These findings and low statistical significance (~25%) demonstrate that merging of the three cohorts’ samples did not improve DEG discovery.

**Figure 3:**
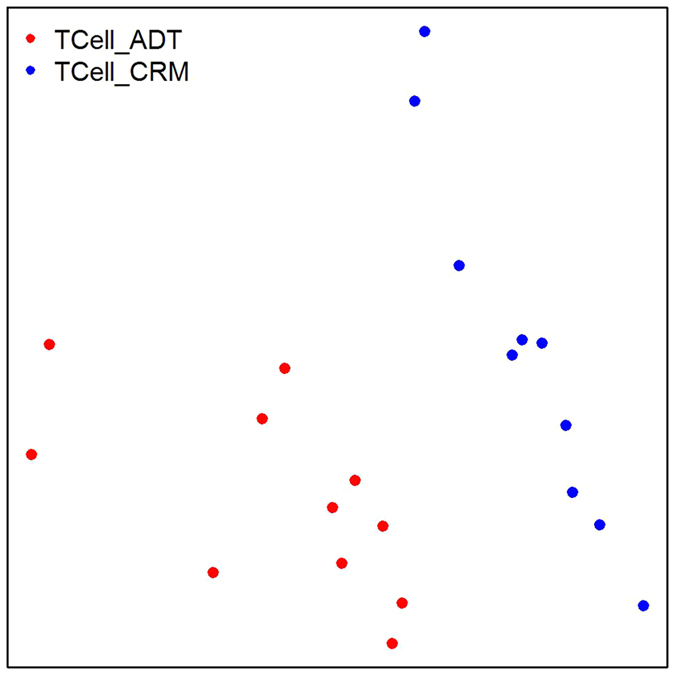
Multidimensional scaling analysis plot for the comparison of ADT (red) and CRM (blue) samples from RNASeq on CD4+ T cells. Euclidean distance was used to measure between samples dissimilarities over gene expression values. Two clusters are formed clearly separating the two groups of JIA patients (TCell_ADT versus TCell_CRM).

The inability to crisply identify DEG for the comparison of treated and remission JIA subjects using RNASeq data from PBMC is likely due to due to mixed transcriptomic signals that emerge from the heterogeneous cell types of PBMC. To test this possibility, we performed MDS analysis using RNASeq data from CD4+ T cells from an independent cohort of JIA samples (11 TCell_ADT and 10 TCell_CRM). We observed two well separated clusters representing children with active, treated disease and treated and children in remission, as shown in Fig. 3. This finding recapitulates what we have seen from RNAseq studies of neutrophils^11^.

### Transcriptomic comparisons and two time points

**Figure 4:**
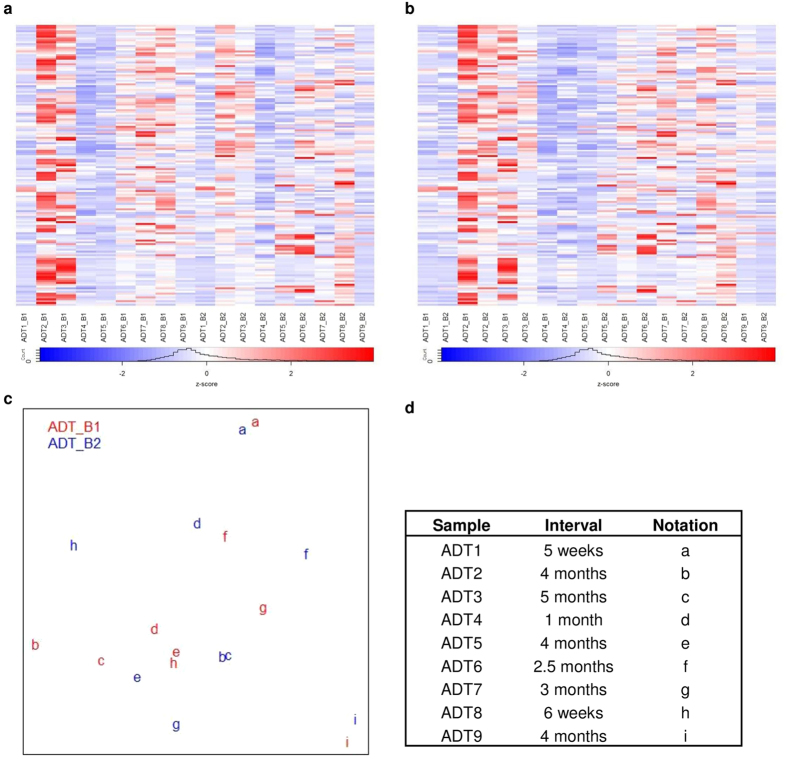
Heatmap of top 1% genes with the largest fold change calculated by voom after sample weighting over normalized gene expressions for cohort B ADT samples. (**a**) Heatmap columns are sorted by time point (B1, B2), (**b**) Heatmap columns are sorted by pair sample of two time points. Rows of heatmap represent gene expression values and column shows sample label (patientID_timePoint). (**c**) Multi dimensional scaling (MDS) plot of gene expression for cohort B ADT samples, red is sample of first time point and blue indicates second time point sample, same subject is represented by identical letter. (**d**) Information of ADT samples corresponding to their intervals across two time points and their notations in MDS plot. Majority of pair samples are alike in their transcription levels despite their medication time course. No significant gene could used to classified ADT samples by medication time course and majority of the identical sample pairs display similar transcription patterns.

Treatment response in JIA is likely to be a continuous process; patients do not suddenly move from active disease to remission in a single step. We thus hypothesized that our inability to distinguish ADT and CRM might be due to the fluid nature of “active disease” and “remission” as treatment response evolves (over the course of active disease) or becomes more established (over the course of remission). We therefore analysed the transcription profiles of PBMC from the patients in cohort B. We first examined the ADT samples over two time points (the range between samples was 5 weeks to 5 months, Supplementary Table 3) to identify transcriptome differences over this time course. MDS analysis showed that the ADT samples randomly scattered and did not cluster based on time points (Fig. 4c). Similarly, when we used voom with sample quality weighting to identify DEG comparing ADT_B1 and ADT_B2, we found 6 DEG at a threshold of p-value ≤0.05 and FC ≥2. Furthermore, few DEG were identified using four additional DEG discovery tools (Supplementary Table 4). Thus, we were unable to attribute the inability of PBMC expression signatures to distinguish ADT and CRM samples to the dynamic nature of treatment response over time; ADT samples collected from the same patients at different time points did not segregate earlier from samples. It is important to point out that the collected ADT samples were heterogeneous in many ways. For example, there were varied intervals across two time points, and the ADT subjects were on different medications at the time the samples were taken (although each individual patient was on the same medications at the time the ADT_B1 and ADT_B2 samples were taken): (i) MTX (n = 4); (ii) MTX and etanercept (n = 3); (iii) MTX and infliximab (n = 1). These different medications likely add to the heterogeneity of the transcriptomic landscapes. At the same time, expression-based biomarkers for “active disease” aimed at guiding treatment decisions will only have clinical utility if they can be used to identify that state regardless of medication or the timing of the sampling.

**Figure 5:**
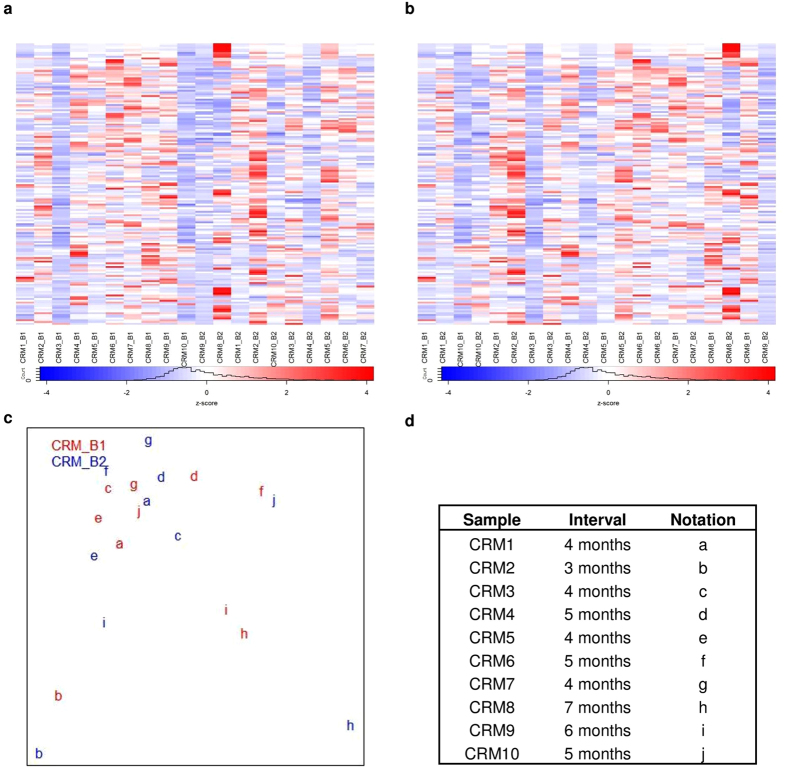
Heatmap of top 1% gene with the largest fold change calculated by voom after sample weighting of normalized gene expressions for cohort B CRM samples over two time points. (**a**) Heatmap columns are grouped by time point (B1, B2), (**b**) Heatmap columns are grouped by pair sample of two time points. Rows of heatmap represent gene expression values and column shows sample label (patientID_timePoint). (**c**) Multi dimensional scaling (MDS) plot of normalized gene expression for cohort B ADT samples, red is sample of first time point and blue indicates second time point sample, same subject is represented by identical letter. (**d**) Information of cohort B CRM samples corresponding to their intervals across two time points with their notations in MDS plot. Paired sample are alike in their transcriptions except CRM6, CRM8, CRM9, CRM10, those samples have interval at least 5 months. None of the top 1% genes of the largest fold change shows distinct clear separation between two time points in CRM samples.

We next sought to determine whether the remission (CRM) samples might be distinguishable based on their timing of collection. We examined transcriptomic dynamics of cohort B JIA patients who have achieved remission on medication over two time points (interval 3 to 7 months). We plotted MDS over gene expressions to preview the cohort B CRM sample distribution. No distinct clusters were observed among the CRM_B samples from the two time points (Fig. 5c). Based on MDS inspection, we saw larger distances between paired samples distinguishing subjects with at least 5 months between samples (CRM6, CRM8, CRM9, CRM10, Fig. 5c) as compared to those paired samples where the interval between sampling was 3–4 months (CRM1, CMR2, CRM3, CRM5, CRM7, Fig. 5c). These findings suggest that the CRM state may evolve over time, and that the later samples may represent a more firmly-established state of remission. However, the heatmaps in Fig. 5 demonstrate that, even though expression patterns allow general categorization of the groups, inter-subject variability makes it impossible to identify one or more genes whose expression levels might serve as candidate biomarkers to test as reliable and unambiguous identifiers of earlier versus more established remission.

We next reduced sample variability using the voomWithQualityWeights function of the limma R package prior to DEG discovery. This analysis resulted two and one DEGs identified before and after the sample quality weighting procedure, with no better performance obtained by the additional four methods for DEG discovery (Supplementary Table 4). Taken together, the paired sample data do not support the conclusion that evolving expression dynamics contribute to the inability to distinguish ADT and CRM from PBMC RNASeq expression profiles. Furthermore, although we observed modest dynamic changes in the CRM state based on the interval between samples, inter-subject variability was too great to select candidate biomarkers that might identify well-established remission and therefore identify children who might safely come off their medications.

### Transcriptomic comparison between JIA patients and healthy controls

Although we were unable to identify biomarkers from PBMC expression data that might be useful in guiding therapy (e.g., by unambiguously identifying remission and distinguishing it from active disease), there are other clinical arenas where biomarkers might be useful. For example, a simple diagnostic biomarker would be a significant advance for the field, as broadly used screening tests such as antinuclear antibody and IgM rheumatoid factor assays have little clinical or diagnostic utility in children^29^. We therefore attempted to examine transcriptomic differences between JIA patients with active, untreated disease (ADU) and heathy controls (HC) with the aim of identifying transcripts that might crisply distinguish one group from the other. First, we performed MDS analysis to determine sample distributions for ADU versus HC. We found that cohort A samples randomly scattered with no distinguishable clusters (Supplementary Fig. 1a), and we therefore chose to focus on cohort C samples for the ADU versus HC comparison. MDS analysis identified outlier samples in cohort C that might hinder DEG discovery, and we therefore removed two ADU_C samples and one HC_C sample before DEG analysis. We used voom with sample quality weighting. This procedure resulted in better classification, with more DEGs identified, 53 versus 92 after outlier removal (Supplementary Fig. 2a,d). We also observed an improved significance level of DEG using this approach, a minimum adjusted p-value of 0.002 as compared to the full sample set which showed a significance level of 0.1685 or higher. However, as demonstrated in Supplementary Fig. 2b–f, although HC and JIA samples crisply segregate on hierarchical clustering and heatmaps, there was still considerable inter-subject variation among both ADU and HC samples, interdicting the selection of one or even a panel of genes whose expression levels might serve as candidates from which to develop reliable diagnostic biomarkers. Furthermore, we were unable to replicate the FC (evaluated by voom with sample quality weighting) for the 92 DEGs using the cohort A ADU versus HC expression data. Pearson correlation of FC for those 92 DEGs (identified using the cohort C data only) between cohort A and C yielded an insignificant correlation of 0.3.

### Transcriptomic comparison of JIA patients before and after therapy

We next examined whether there might be other uses for PBMC transcriptional analysis in JIA, for example, understanding disease mechanisms or mechanisms of treatment response/non-response. For example, very little is known about how transcriptomes are re-organized in early therapy of JIA patients, although our work^30^ and the work of others^31^ has suggested that the first four months after initiating therapy are critical in determining outcome. It is useful to compare at baseline (before treatment begins) and early therapy of JIA individual transcription profiles to identify transcripts that may be crucial to determining treatment response. By capturing transcriptional dynamics as JIA patients move from the untreated to treated stage, we might use the unique expression profile of patients over these two stages to predict responsiveness to a particular treatment regimen. For example, at baseline, JIA patients who respond to MTX may be indistinguishable from those who won’t, but the distinctions might become apparent after therapy-induced transcriptional alterations.

For this analysis, first we previewed sample distributions of ADU and ADT samples from cohort A and cohort C, no time course information for those ADT subjects. MDS analysis showed that most of the ADU samples from cohort A formed sub-cluster within ADT samples (Supplementary Fig. 1b), suggesting that varied transcription dynamics in early therapy might due to individualized treatment responses. Overall, we saw heterogeneous expression patterns in the comparison of ADU and ADT samples (Supplementary Fig. 1b). We observed better separation between ADU and ADT when we used only samples from cohort C (Supplementary Fig. 3a).

We next ran DEG analysis on two cohorts (A and C) independently using voom with sample quality weighting. This procedure resulted in the identification of 12 and 131 DEG (at threshold of p-value ≤0.01, FC ≥2) for cohort A and C respectively, with no common DEG between the two cohorts. As with the previous analyses, within group sample heterogeneity is one of the strong challenges in discovering candidate biomarkers (Supplementary Fig. 3b), as demonstrated by the interweaving patterns of gene expression for ADU_A and ADT_A samples in the MDS spreadsheet (Supplementary Fig. 1b). Furthermore, the mixed cell population of PBMC poses another level of heterogeneity that challenges DEG analysis and thus our ability to either develop mechanistic insights into treatment response or develop candidate biomarkers to predict treatment response.

Follow this, we investigated the differences in transcription profiles between treated JIA patients^32^ and healthy controls as a way of determining whether PBMC RNASeq data might yield insights into transcriptional response that might either lead to identification of useful biomarkers or provide insight into mechanisms of therapeutic response. For example, Domoroz *et al.* demonstrated that ADT subjects could be grouped by treatment response such as partial, full, or non-responsive based on microarray analyses of peripheral blood buffy coat samples^33^. Using MDS plot visualization, we found indistinguishable clusters of samples between ADT_A and HC_A (Supplementary Fig. 1c), but observed two distinct clusters formed by samples from ADT_C and HC_C (Supplementary Fig. 4a,d). We then focused on DEG analysis for ADT_C versus HC_C, and found 119 DEGs using voom with sample quality weighting after outlier removal (adjusted p-value ≤0.01 and FC ≥2). A heatmap of these DEGs indicates that FC for most of the DEGs were likely to be dominated by two or three HC_C samples (Supplementary Fig. 4f). As with previous analyses, we were unsuccessful, in attempting to replicate our findings by comparing FC (base on voom with sample quality weighting calculation) for ADT_A versus HC_A for DEGs discovered using cohort C data (correlation 0.44).

Finally, we sought to determine whether PBMC transcriptomes generated by RNASeq might give us insights into the biology of remission by comparing transcriptional profiles of JIA individuals who achieved remission on medication (CRM) with healthy controls. MDS and hierarchical clustering analysis resulted in two clusters separating samples from CRM_C and HC_C (Supplementary Fig. 5a,d) but no clear separation for CRM_A and HC_A samples (Supplementary Fig. 1d). As with previous analyses, we implemented voom with sample quality weighting for the CRM_C versus HC_C comparison, and identified 94 and 101 DEG before and after outlier removal respectively. Based on the heatmap visualization of DEGs’ transcription levels, we observed that a small number of HC_C samples heavily influenced the FC for majority of the DEGs (Supplementary Fig. 5c,f). From the 101 DEGs, we did not identify any gene of statistical significantly difference between CRM and HC in cohort A.

### Reliability of the differentially expressed genes analysis pipeline

A series of DEG analysis for all pair group comparisons showed the difficulty of developing reliable biomarker candidates or developing mechanistic insights from PBMC RNASeq data. As a final quality control measure, we tested the reliability of our DEG analysis pipeline. To do this, we downloaded raw sequencing reads from the Smchd1 experiment (GEO accession number GSE64099)^21^, and repeated the identical analysis workflow we had applied in our PBMC RNASeq data (see Methods for details). DEGs found using voom with sample quality weighting showed 80% concordance with respect to the DEG list reported^21^ using the same DEGs discovery tool (voom with sample quality weighting). The differences may reflect the fact that Liu *et al.* adopted a different method for reads mapping (Subread version 1.10.5^34^) and gene expression measurement (featureCounts procedure^35^). This high concordance rate (80%) of DEG list indicates that our DEG discovery pipeline is correct.

## Discussion

When genome-wide expression technologies were introduced, they were heralded as a promising means through which biomarkers might be developed in a broad variety of diseases, including rheumatic disease such as rheumatoid arthritis and JIA^36^. While there has been some success in developing biomarkers to predict treatment response in rheumatoid arthritis^4^,^5^, challenges remain in adapting RNASeq data for clinical purposes^37^. At the present time, expression-based biomarkers developed from microarray technologies have not emerged for the rheumatic diseases of childhood. Ideally, biomarker development ought to begin by the identification of potential candidates, preferably using relatively small sample sizes in the interest time and cost, proceed with replication in a larger independent cohort, followed by validation in a blinded cohort in which the phenotype of the tested sample is unknown.

One of the important issues in developing biomarkers for clinical adaptation is the selection of appropriate cells or tissues from which to identify candidates and then develop biomarkers. PBMC remain attractive candidates because they are easy to obtain and simple to prepare. However, PBMC carry significant potential disadvantages from the standpoint of developing expression-based biomarkers. The most significant of these disadvantages is the fact that PBMC represent a heterogeneous cell population, including cells of both myeloid and lymphoid linages, with each of these linages also containing distinct sub-populations. At the same time, transcriptomes show considerable cell-type specificity, reflecting the specific functions of each cell type and subtype. Thus, while there are some broad commonalities that both ENCODE and the Roadmap Epigenomics projects have identified in the transcriptomes of peripheral blood cells^14^,^15^, it is unclear that these commonalities are sufficient to reduce the inherent noisiness of gene expression data derived from heterogeneous cell populations.

We generated RNASeq data from PBMC using 3 independent patient cohorts in an attempt to determine whether we could identify biomarker candidates that would assist in either disease staging (i.e., identify patients on therapy who had achieved remission [CRM] and distinguish them from children who still had active disease [ADT]) or in diagnosis (comparing untreated children [ADU] and healthy controls). Our data demonstrate that adding an additional layer of variability in the use of a mixed cell population compounds the already-existing challenges in the use of RNASeq data as a first step in identifying clinically useful biomarkers. These challenges include both technical and biological issues.

The technical issues that may introduce variability in RNASeq data have been discussed in detail recently^38^. They encompass RNA preparation, the generation of cDNA, library preparation, and the sequencing reactions themselves. Our data demonstrate these issues well, as there were discernible differences in the quality and depth of the sequencing reactions between cohorts A and C. These differences very likely contributed to our inability to corroborate disease-state-specific DEG identified in the C cohort in the A cohort samples. Technical variability might be reduced by sequencing a larger number of samples and excluding poor-quality samples from the analysis^39^,^40^, but this adds considerably to the expense of what ideally would be a first-stage screening procedure to identify biomarker candidates that would then be validated by quantitative rtPCR.

Another issue that the field faces is the clinical and biological heterogeneity of the disease entity we call “polyarticular JIA”. Patients with this disease display a broad range of ages at presentation (typically age 1 to 15 years), differences in disease severity (e.g., in the number of joints affected) considerable differences in their responses to specific therapies, differences in autoantibody expression, and differences in the rates of specific complications such as uveitis. These phenotypic differences are accompanied by well documented biological differences^41^ that can be discerned when multiple analyses (including peripheral blood gene expression) are used to characterize patients. Indeed, one potentially useful application of PBMC RNASeq in the context of JIA may be to more crisply identify heterogeneous patient sub-groups, which was not the aim of the current study.

The heterogeneity of the patient population is not the only issue. Recent studies have found considerable differences in gene expression in healthy individuals that are linked to genetic variance^32^,^42^. This finding was reflected in our own data, particularly Cohort C, where expression levels of 2 healthy individuals accounted for most of the differences between patients and healthy controls (Supplement Figs 4f and 5f). Many of the issues regarding heterogeneity could be ameliorated (but not eliminated) by using more homogeneous patient populations. Doing so, however, reduces the chances of identifying clinically-useful biomarkers that that will be applicable to all patients or reliably distinguish children with the disease from healthy controls regardless of age, ethnicity, or other variables that might impact gene expression.

Taken together, multiple analysis approaches failed to crisply separate ADT and CRM samples from RNASeq of PBMC. This could be a real biological phenomenon, reflecting the fact that the ADT and CRM states are too similar in their transcriptome properties to be distinguished. However, it is equally plausible that PBMC, being a heterogeneous mixture of cell types, produce a wide range of signals that contribute to a noisy transcriptional landscape. The heterogeneity of the sample is further complicated by the known heterogeneity of the patient population designated, “polyarticular JIA”^41^. At the same time, the clinical utility of biomarkers in JIA will depend on whether they are broadly applicable to this hreterogeneous population; the field needs to be able to identify “active disease” whether the sample is taken 3 weeks or 3 months after treatment is started, and remission whether the remission state has been stable for 3 or 9 months. In this respect, the current study was somewhat reassuring. Samples taken from children in Cohort B with active disease were indistinguishable from samples taken 5 weeks to 6 months later when these same children still had active disease. Similarly, remission (CRM) samples from Cohort B showed no tendency to segregate based on the timing of sampling. These similarities in expression profiles were maintained even for children on different medications. However, MDS analysis observation suggest that CRM patients may exhibit larger differences in their transcriptome profiles with longer time intervals, and, thus, it is possible that biomarker candidates for sustained remission may emerge from studies of larger groups of samples.

We should point out that neither subject heterogeneity (patients or controls) nor sample sizes interdict proper JIA classification (e.g., active disease versus remission) when relatively homogeneous cell populations are used in RNASeq studies. In the current study, we demonstrate that we can easily classify ADT and CRM samples based on MDS analysis of RNASeq data from CD4+ T cells. Our group has also recently shown that RNASeq expression patterns in neutrophils crisply identify children with untreated JIA and those in remission^11^. Furthermore, neutrophil RNASeq distinguished children with JIA from children with cystic fibrosis, demonstrating that the JIA neutrophil expression patterns were not simply a non-specific indication of chronic inflammation in soft tissues. It is reasonable to assume that equally crisp delineations of disease phenotypes might be obtained with a more homogeneous sample of lymphoid cells, e.g., CD4+ T cells or FoxP3+ cells. However, even if candidate biomarkers were identified in these populations, they would not be easy to introduce into clinical use. Separating and analyzing T cell subsets would be expensive, and, as our data show, detecting such subset-specific biomarkers without prior isolation of specific subsets would be difficult against a background of whole blood or even PBMC expression.

At the present time, conventional, serum based biomarkers will probably continue to be the most reliable approach for monitoring therapy and providing a basis for clinical decision-making. For example, Gerss *et al.* have shown that serum level of S100 proteins and high-sensitivity C-reactive protein assays can distinguish JIA patients who will flare within 6 months after coming off therapy from those who will go on to more stable remission^43^.

While ours was essentially a negative study, it carries important findings for the field of pediatric rheumatology and translational research in rheumatology in general. Several large clinical trials in juvenile arthritis are about to be launched, and there is considerable interest in the field in obtaining appropriate clinical samples to elucidate the basic biology of treatment response or non-response.

While PBMC carry many advantages in terms of the ease with which they can be obtained and stored, they are very likely to be limited in what they can tell us in gene expression studies. Our data demonstrate that the heterogeneity of this cell population adds an additional level of variance that already challenges both biomarker development and mechanistic studies aimed at understanding the underlying biology of therapeutic response. These findings should be considered in planning the collection of biological research specimens in the context of pediatric rheumatology clinical trials.

## Materials and Methods

### Samples

We studied children with the polyarticular, rheumatoid factor-negative form of JIA (as defined by international criteria^44^) using three independent cohorts. In the first cohort (denoted as cohort A), we studied 8 children with newly diagnosed untreated disease (ADU_A), 8 children with active disease who had been on treatment with methotrexate ± a TNF inhibitor for periods ranging from 3 weeks to 3 months (ADT_A), 8 children who fit criteria for clinical remission on medication (CRM_A)^20^ and 8 healthy controls (HC_A) were recruited from the University of Oklahoma and Women and Children’s Hospital of Buffalo General Pediatrics Clinics. HC did not have underlying systemic inflammatory diseases and were excluded if they had fever within the previous 36 hours, were taking oral glucocorticoids, or had a BMI of ≥30 (since obesity is associated with an indolent inflammatory response). For the second cohort (cohort B), we studied 19 patients with active disease on treatment (ADT_B, n = 9) and in clinical remission on medication (CRM_B, n = 10). Each child of cohort B was studied on at least 2 occasions. For ADT_B patients (9 pairs), which we designate ADT_B1 and ADT_B2, blood was taken on 2 occasions 5 weeks to 6 months apart (mean = 2.9 months). The CRM_B samples (10 pairs) were taken 3 to 7 months apart (mean = 4.7 months) and designated CRM_B1 and CRM_B2. Patients in cohorts A and B were recruited from the University of Oklahoma Health Sciences Center pediatric rheumatology clinic. In the third cohort we studied 8 in each of the three different JIA states children with JIA as well as for HC subjects (denoted as ADU_C, ADT_C, CRM_C and HC_C). 16 patients from cohort C were recruited from the Seattle Children’s Medical Center, 8 healthy controls of cohort C were recruited from Women and Children’s Hospital of Buffalo General Pediatrics Clinics. Patient characteristics for each cohort are provided in Supplementary Table 3.

In addition to 3 cohorts data on PBMC RNASeq, to supplement our data analysis we incorporated CD4+ T cells RNASeq data from an independent cohort of JIA patients that contained 12 ADT and 10 CRM samples (denoted as TCell_ADT amd TCell_CRM respectively). This set of JIA patients was recruited from Women and Children’s Hospital of Buffalo General Pediatrics Clinics. ADT and CRM patients were all on treatment with both etanercept and methotrexate. CD4+ T cells were prepared from PBMC using negative selection procedures.

The research protocols for this study were approved by the institutional review boards (IRBs) of the University at Buffalo, University of Oklahoma Health Sciences Center and Seattle Children’s Medical Center. The research methods were carried out adhering to the approved IRBs protocols. Informed consent was obtained from the parents of all patients and healthy controls prior to obtaining specimens. In order to accomplish the goals of this study, it was necessary to study the same patient on two occasions at the same disease stage (biological replication) as well as to access technical reproducibility by examining samples from a completely independent patient cohort. To increase the rigor and general applicability of these studies, cohorts A, B and C were sequenced in two different sequencing facilities. Developing biological replicates was obviously not possible for children with active, untreated JIA, as therapy starts as soon as possible after a diagnosis. Similarly, we have found that it is difficult to get more than one sample from a given child who has achieved the inactive disease as defined by the Wallace criteria^20^, as these children have frequently achieved the CRM state by the time of their next regular clinic visit. However, children with active disease typically maintain that state for weeks or even months, even when therapy ultimately results in the attainment of CRM. Similarly, the CRM state can be sustained for many months in children, even though true clinical remission (maintenance of a disease-free state for 12 continuous months after medications are discontinued) is rare^20^.

### Patient treatment stages

We used the Wallace criteria^20^ to assign disease activity or treatment stage to each sample. Children whose samples were assigned to the active, treated disease category^32^ all had synovitis, assessed by the presence of warmth and synovial proliferation, in at least one joint. Children whose samples were assigned to the CRM category had achieved clinically inactive disease (CID) status: (i) no joints with active arthritis; (ii) no fever, rash, serositis, splenomegaly or generalized lymphadenopathy attributable to JIA; (iii) no active uveitis; (iv) ESR in the normal range in the laboratory where tested; (v) a physician’s global assessment of disease activity score of 0 and (vi) morning stiffness <15 minutes. CRM patients had maintained the CID state for at least 6 continuous months.

### RNA sequencing sample preparation

For both PBMC and CD4+ T cell RNASeq sample preparation, whole blood was drawn into 10 mL citrated Cell Preparation Tubes (Becton Dickinson, Franklin Lakes, NJ, USA). For PBMC, cell separation procedures were started within one hour from the time the specimens were drawn. PBMC and CD4+ T cells were separated from granulocytes and red blood cells by density-gradient centrifugation. PBMC were then immediately placed in TRIzol™ reagent (Invitrogen, Carlsbad, CA, USA) and stored at −80 °C. For CD4+ T cell, cells were isolated using negative selection from each sample using StemSep™ Human CD4+ T Cell Enrichment Kit (STEMCELL Technologies Inc., Vancouver, Canada).

Total RNA was extracted using TRIzol™ reagent according to manufacturer’s directions. RNA was further purified using RNeasy MiniElute Cleanup kit including a DNase digest according to the manufacturer’s instructions (QIAGEN, Valencia, CA). RNA was quantified spectrophotometrically (Nanodrop, Thermo Scientific, Wilmington, DE) and assessed for quality by capillary gel electrophoresis (Agilent 2100 Bioanalyzer; Agilent Technologies, Inc., Palo Alto, CA). cDNA libraries were prepared for each sample using the Illumina TruSeq RNA Sample Preparation Kit by following the manufacture’s recommended procedures. Libraries were sequenced using 100 basepair (bp) paired-end reads on Illumina HiSeq 2500 instruments. Library construction and RNASeq were performed at the Columbia University Genome Center (cohort A and C) or at the University at Buffalo Genomics and Bioinformatics Core (both replicates of cohort B and CD4+ T cells samples).

### RNA sequencing data analysis

Raw paired-end reads were aligned to the human reference genome hg19 downloaded from the University of California Santa Cruz Genome Bioinformatics Site, UCSC (http://hgdownload.soe.ucsc.edu/goldenPath/hg19/chromosomes/) using TopHat version 2.0.13^45^. Mapping analysis resulted an average of 42.2 million mapped reads per sample, with an average mapping rate of 87.2% over all samples from three cohorts (Supplementary Table 5).

Prior to downstream analysis, we carried out a series of quality control (QC) checks on the RNASeq reads. Using FASTQC tool (http://www.bioinformatics.babraham.ac.uk/projects/fastqc/), we conducted QC on raw sequenced reads. Supplementary Table 6 summarizes the number of raw sequences grouped by cohorts. Cohort C samples had the least degree of variation across samples in terms of number of raw reads, while samples from cohorts A and B had coefficients of variance (COV) greater than 10%, relatively high between-sample variances in term of read depth. Next, we examined the read distribution by genomic features, i.e., exonic, intron, transcription start site^32^ and transcription end site^12^ for each sample using RSeQC tool version 2.3.6^46^. We found that an average of 78% of reads were from exonic regions across all samples in all three cohorts (Supplementary Table 7). A minimum 70% exonic rate can be used to dismiss the possibility of DNA contamination in RNASeq library preparation, and, thus, our entire set of samples passed the exonic rate QC check. Following this, we checked for coverage uniformity to ensure absence of read depth bias in 3′ or 5′ positions by calculating read coverage for each nucleotide using geneBodyCoverage.py module in RseQC. We did not detect any sample from the three cohorts with 3′ (5′) bias, but two samples from cohort A displayed tendencies toward 5′ bias (Supplementary Fig. 6). Lastly, we a conducted GC content QC check to ensure that the GC distribution across the sequenced reads from all samples fell within the expected distribution. We saw a normal distribution of GC content, with peak at 40–50% for all samples, and no outlier sample demonstrating GC content bias was detected (Supplementary Fig. 7).

Gene expression abundance was calculated using the htseq-count function from the HTSeq Python package^47^, with GENCODE v19 gene annotation as reference^48^. Gene expression counts were then used for DEG analysis using the voom and eBayes function from the limma R package^49^ (for simplicity, voom was denoted as DEG analysis unless otherwise mentioned). Voom^50^ estimates mean variance relationships of gene abundances and generates weight for each observation as input to the limma empirical Bayes analysis pipeline through eBayes function. As described by Law *et al.*^50^, voom pre-processes data for empirical eBayes linear modelling^51^ by first transforming gene abundance raw counts to log2 read counts per million (log2CPM). This procedure is followed by linear model fitting to estimate a log2CPM mean-variance relationship that produces residual standard deviations for each gene and fitted values for each log2CPM. For each gene, voom then computes average log2 counts from average logCPM and fitted log2 counts from log2CPM. Next, voom uses Locally Weighted Scatterplot Smoothing (LOWESS) regression^52^ to non-parametrically fit square roots of residual standard deviations as a function of average log2 counts for each gene. The LOWESS fitted curve is used to map square root standard deviations with respect to fitted log2 counts. For gene expression of each sample, the inverse predicted variance is the weight for that observation. eBayes function takes the log2CPM values and the measured weights from voom as input for DEG analysis by performing linear regression on a matrix with rows consisting of gene expression levels, and columns contain samples. The associated regression coefficients, empirical Bayes moderated t-statistics and p-values are used to estimate the significance of the expression changes^53^. The default cutoff of FC and adjusted p-value for defining DEG was 2 and 0.05 respectively. Due to varied characteristics of the transcriptomic data from the different paired groups, we adjusted the threshold for FC and adjusted p-value corresponding to different paired group comparisons according to observations on volcano plots^54^.

We examined paired group sample distribution using MDS analysis over gene expression values, with Euclidean distance measurement for MDS for the top 500 genes of the largest variation between groups; the limma R package plotMDS function was used for this analysis^49^. MDS plot was used for preview on the separation between samples of the compared paired groups. In addition, we visually inspected inter sample variabilities based on gene expression using heatmap plotting from the gplots R package^55^, adopting Euclidean distance measurement and complete linkage methods in hierarchical clustering.

As our purpose for including CD4+ T cells RNASeq data is to supplement the PBMC data analysis, we applied a simplified analysis pipeline onto CD4+ T cells data. Similar to PBMC data, we mapped CD4+ T cell raw sequenced reads to the human reference genome hg19 using TopHat, followed by gene expression abundance calculation using htseq-count. Gene expression abundances of 21 samples (11 TCell_ADT and 10 TCell_CRM) were then used for MDS analysis to determine whether group separations (i.e., TCell_ADT versus TCell_CRM) are possible based on RNASeq expression signatures.

### Reliability of the differentially expressed genes analysis pipeline

We tested the accuracy of the DEG analysis workflow by applying an identical analysis workflow that we used for PBMC RNASeq in SMchD1 experiment data^21^. This RNASeq study aimed to identify genes that were regulated by SMchd1 in lymphoma cell lines of Mus musculus, and the samples consisted of three wild type and four mutant samples. We downloaded raw sequencing reads from the Smchd1 experiment (GEO accession number GSE64099^21^), performed read mapping using TopHat on default settings with respect to the mm10 mouse reference genome (downloaded from UCSC at http://hgdownload.cse.ucsc.edu/goldenPath/mm10/bigZips/chromFa.tar.gz). Gene expression abundance was calculated using htseq-counts on mapped reads using UCSC Mus Musculus mm10 gene annotation, followed by voom, with sample quality weighting for DEG analysis.

## Additional Information

**How to cite this article**: Wong, L. *et al.* Limits of Peripheral Blood Mononuclear Cells for Gene Expression-Based Biomarkers in Juvenile Idiopathic Arthritis. *Sci. Rep.* **6**, 29477; doi: 10.1038/srep29477 (2016).

## Supplementary Material

Supplementary Information

## Figures and Tables

**Table 1:** Number of differentially expressed genes for the comparison between ADT and CRM.

	FC	APV	cohort A	FC	APV	cohort B	FC	APV	cohort C
cuffdiff2	2	0.05	35	2	0.05	26	2	0.05	63
deseq2	1.3	0.10*	1	1.4	0.05	6	1.3	0.10*	3
EBSeq	2	0.05	1	2	0.05	11	2	0.05	71
edgeR	2	0.05	2	2	0.05	31	2	0.05	4
voom	2	0.05*	8	2	0.05*	11	2	0.05*	19
voom+weight	2	0.05*	17	2	0.05*	11	2	0.05*	14

FC: fold change, APV: adjusted p-value, *p-value is used for cutoff.
