# Supplementary material for: Limits of Peripheral Blood Mononuclear Cells for Gene Expression-Based Biomarkers in Juvenile Idiopathic Arthritis
Source: Sci Rep. 2016 Jul 7;6:29477. doi: 10.1038/srep29477 (PMC4935846; doi:10.1038/srep29477)
Supplement: Supplementary Information [file srep29477-s1.pdf]

Supplementary Information

Limits of Peripheral Blood Mononuclear Cells for Gene Expression-Based Biomarkers in Juvenile Idiopathic Arthritis

Laiping Wong[1], Kaiyu Jiang[1], Yanmin Chen[1], Teresa Hennon[1], Lucy Holmes[1], Carol A. Wallace[3], James N. Jarvis[1,2]

[1] Department of Pediatrics, University at Buffalo, Buffalo, NY

[2] Genetics, Genomics, & Bioinformatics Program, University at Buffalo, Buffalo, NY

[3] Department of Pediatrics, University of Washington, Seattle, WA

Email addresses

Laiping Wong: laipingw@buffalo.edu

Kaiyu Jiang: kaiyujia@buffalo.edu

Yanmin Chen: yanminch@buffalo.edu

Teresa Hennon: trussell@buffalo.edu

Lucy Holmes: luholmes@buffalo.edu

Carol Wallace: cwallace@u.washington.edu

James N. Jarvis: jamesjar@buffalo.edu

Corresponding author: James N. Jarvis, M.D., University at Buffalo Clinical & Translational Research Center R8026, 875 Ellicott Street, Buffalo, NY 14203.

Email: jamesjar@buffalo.edu

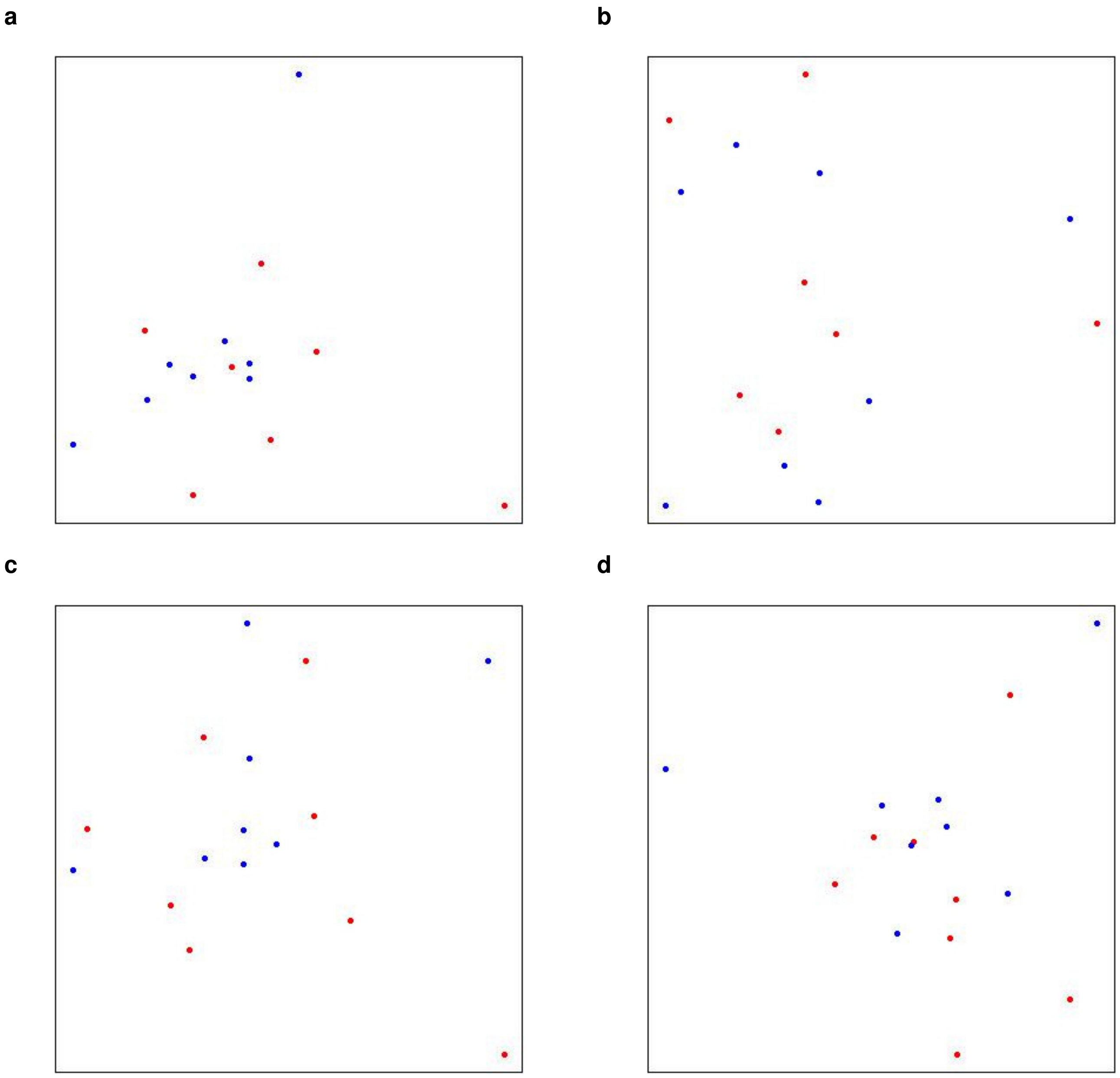

Supplementary Figure 1. Multidimensional scaling analysis plot for cohort A samples for the comparison of (**a**) ADU (red) versus HC (blue), (**b**) ADU (red) versus ADT (blue), (**c**) ADT (red) versus HC (blue), (**d**) CRM (red) versus HC (blue). Samples are randomly scattered with no distinguishable clusters between the observed paired group for all paired group comparison in cohort A.

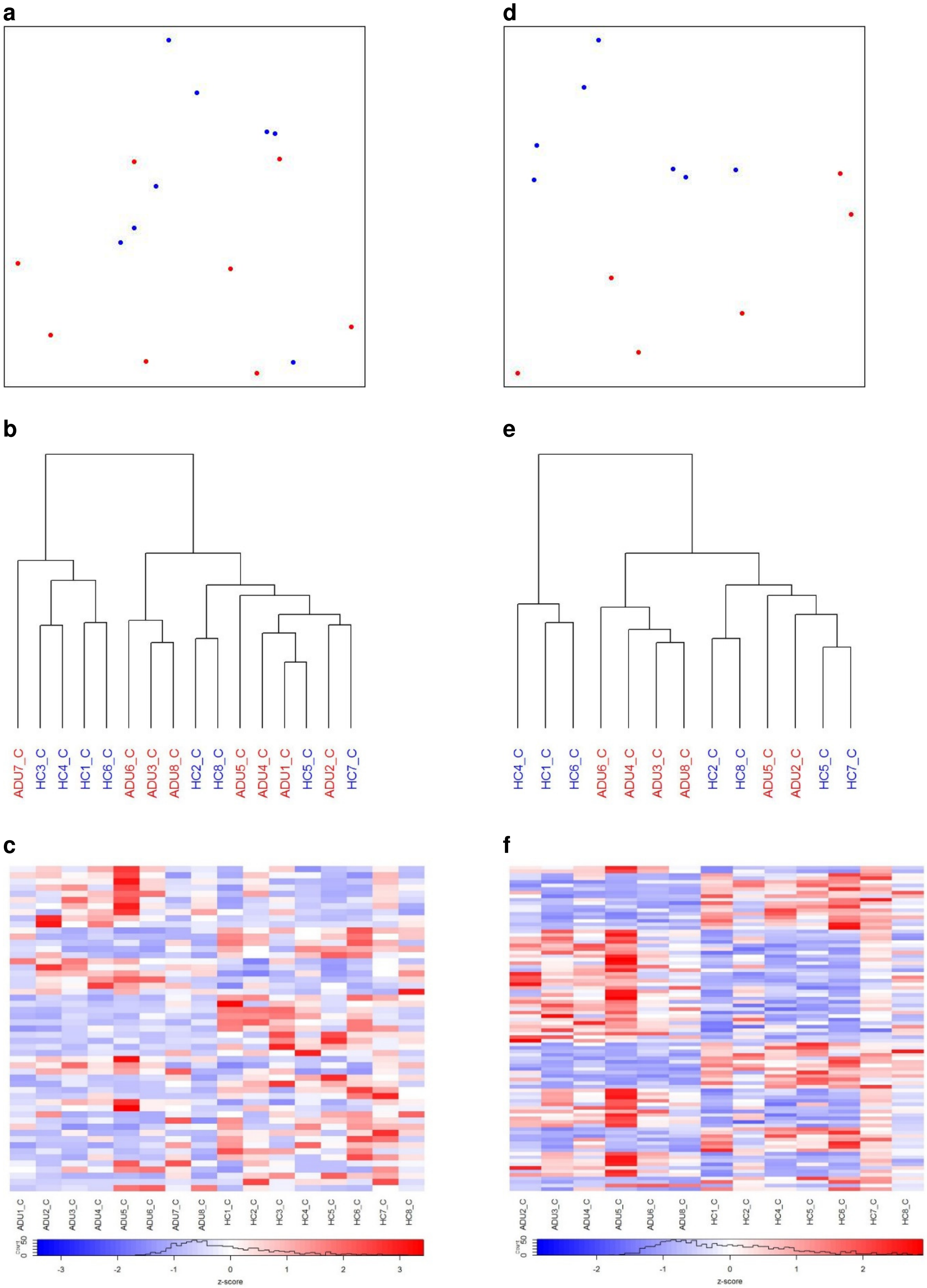

Supplementary Figure 2. Comparison of ADU_C and HC_C. Differentially expressed genes (DEG) analysis before and after two ADU_C and one HC_C samples removal are represented by left and right column respectively. (**a**, **d**) Multidimensional scaling plots over gene expressions show two clusters representing ADU_C and HC_C samples after outlier removal. Hierarchical clustering before (**b**) and after (**c**) outlier removal exhibits distinguishable sub-clusters formed corresponding to samples of ADU_C and HC_C group. Heatmap for (**c**) DEG using voom only and (**f**) DEG using voom with sample quality weighting that discovered more DEG.

a **b**

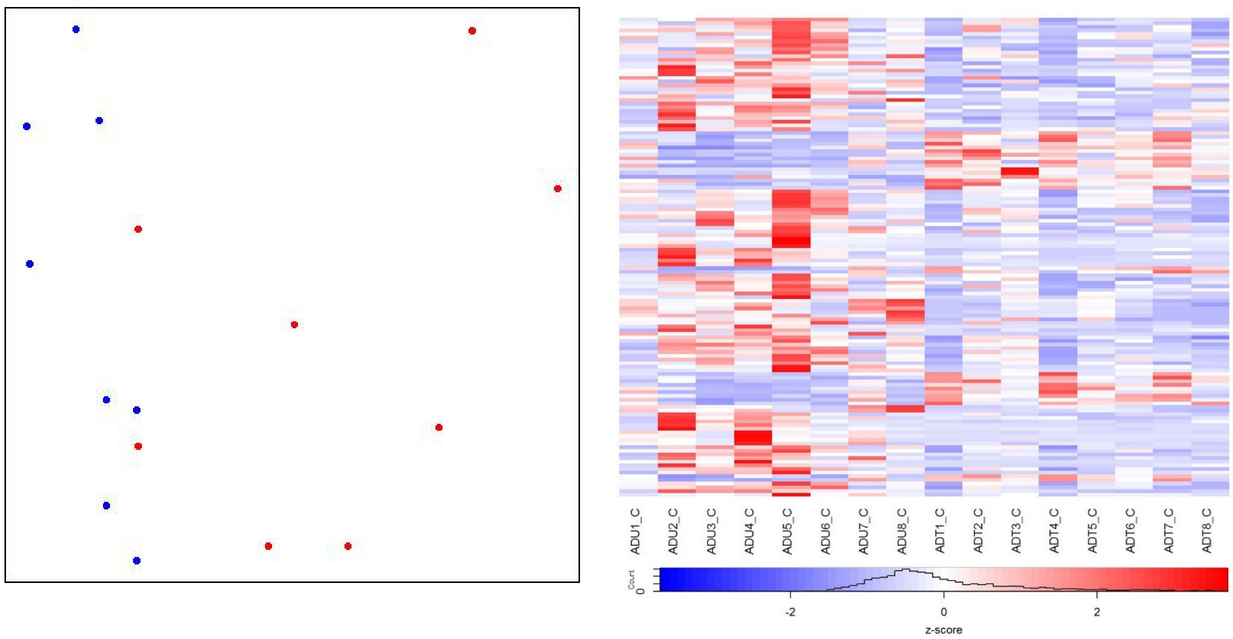

Supplementary Figure 3. Comparison of ADU_C and ADT_C. (**a**) Multidimensional scaling plots over gene expressions show two separable clusters representing ADU_C and ADT_C samples. (**b**) Heatmap for differentially expressed genes at p-value≤0.01 and fold change≥2, detected using voom with sample quality weighting.

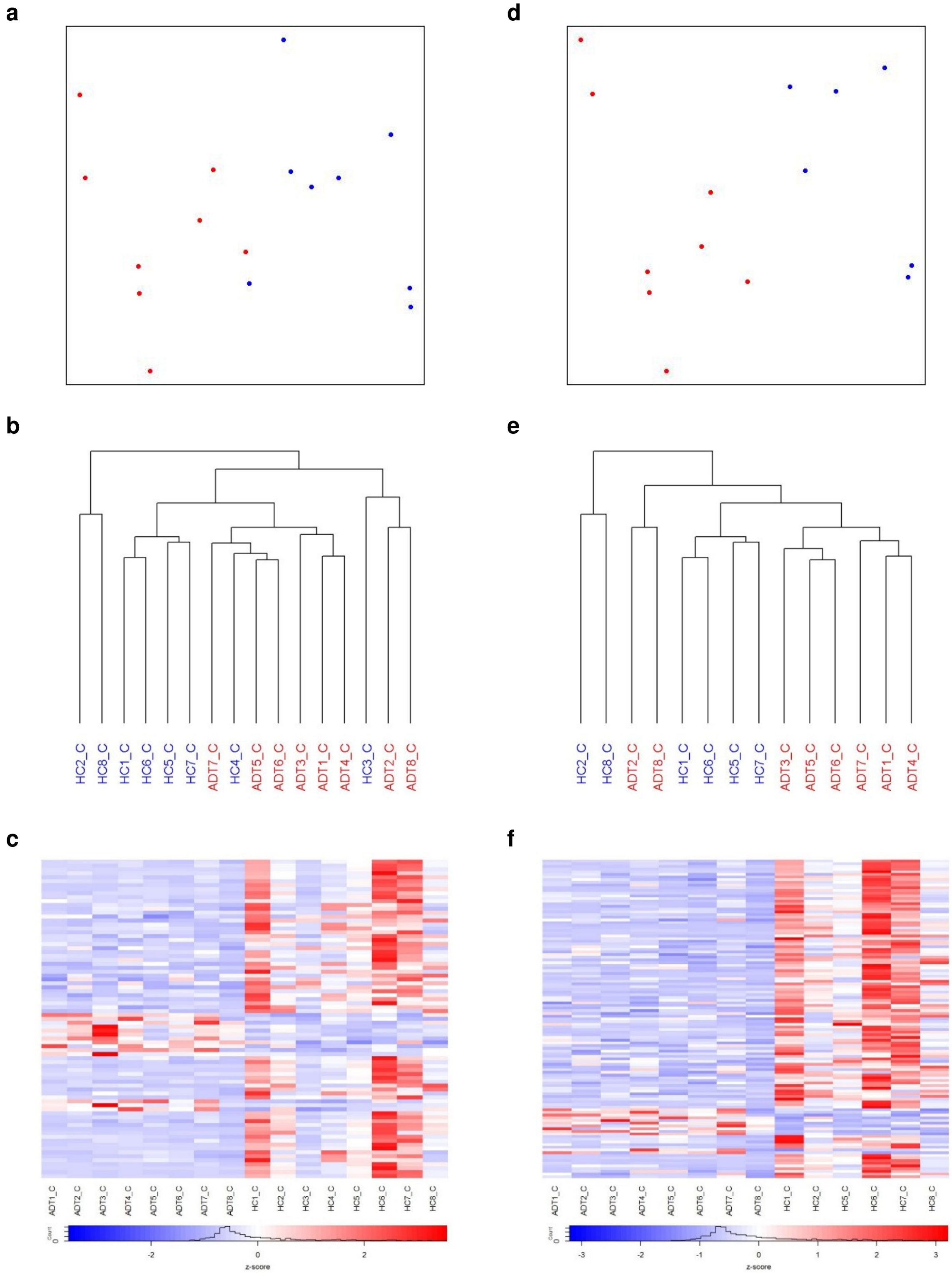

Supplementary Figure 4. Comparison of ADT_C and HC_C. Analysis before and after two HC_C samples removal are represented by left and right column respectively. (**a**,**d**) Multidimensional scaling plots over gene expressions show two well separable clusters representing ADT_C and HC_C samples after outlier removal. (**b**) Hierarchical clustering displays two HC_C samples (outliers) grouped into ADT_C cluster and (**c**) Hierarchical clustering after outlier removal exhibits distinguishable sub-clusters formed corresponding to ADT_C and HC_C group. Heatmap for (**c**) 81 DEGs before removing outliers and (**f**) 119 DEGs after removing outliers with adjusted p-value≤0.01 and fold change≥2, detected using voom with sample quality weighting.

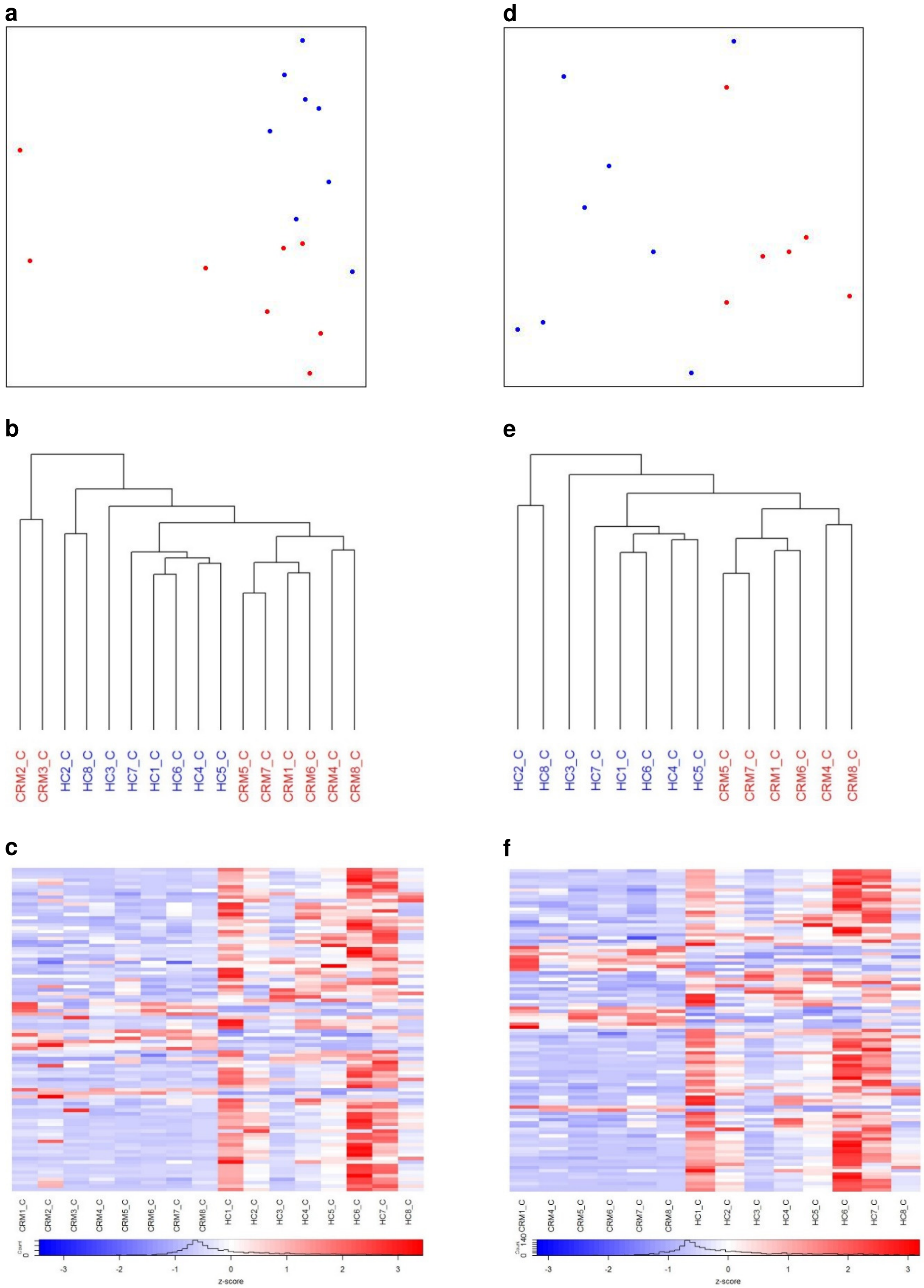

Supplementary Figure 5. Comparison of CRM_C and HC_C. Analysis before and after two CRM_C samples removal are represented by left and right column respectively. (**a**, **d**) Multidimensional scaling plots over gene expressions show two well separable clusters representing CRM_C and HC_C samples after outlier removal. Hierarchical clustering (**b**) before and (**e**) after outlier removal. Heatmap for (**c**) 94 DEGs before outlier removal, (**f**) 101 DEGs after outlier removal at threshold of p-value≤0.01 and fold change≥2, detected using voom with sample quality weighting.

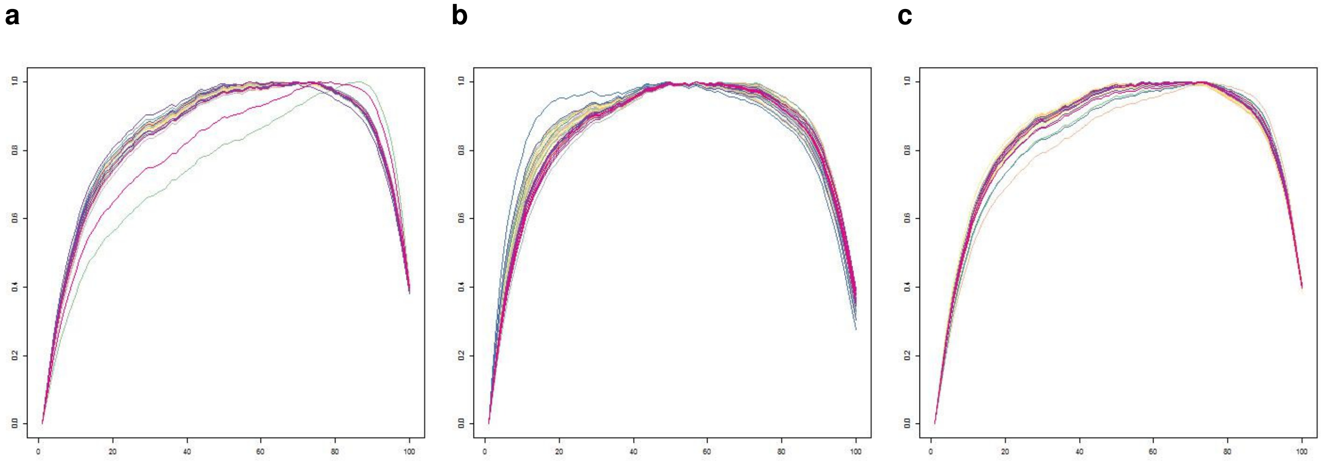

Supplementary Figure 6. Gene body coverage for samples from (**a**) cohort A, (**b**) cohort B and (**c**) cohort C. Vertical axis represents read coverage for each nucleotide position (horizontal axis) of all genes normalized to 100 bins and each sample is showed by colored line. Uniformity in gene body coverage amongst samples of all cohorts, as note that two cohort A samples display 5’ bias tendencies.

a b c

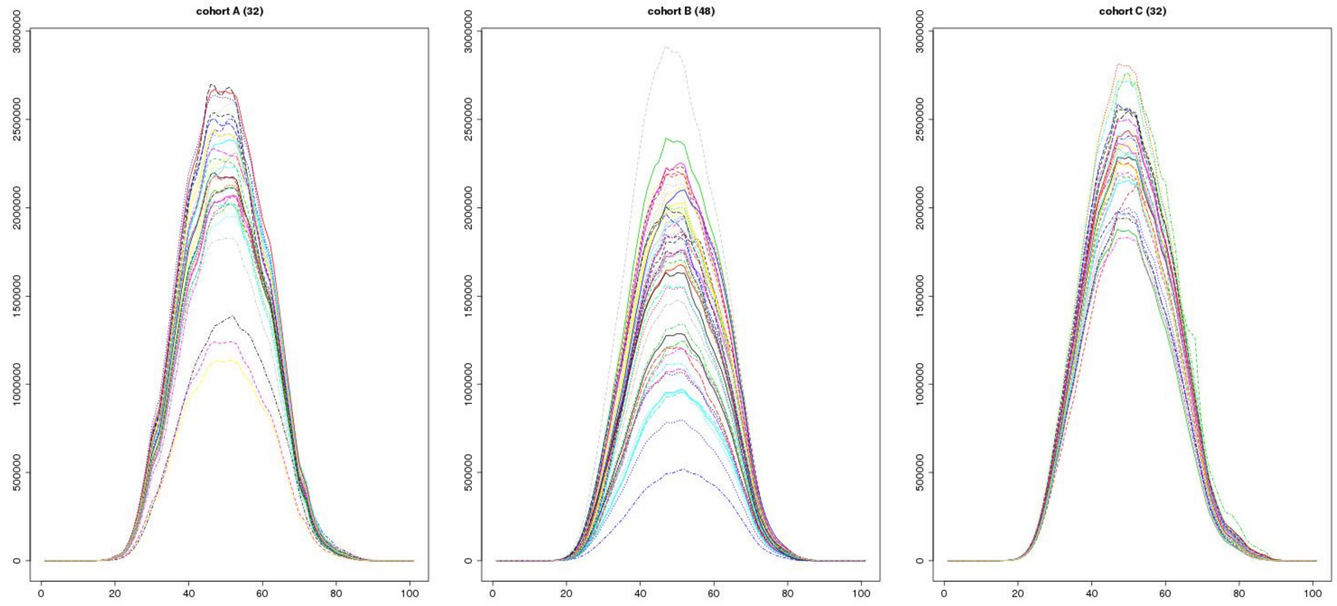

Supplementary Figure 7. GC content distribution for samples from (**a**) cohort A, (**b**) cohort B and (**c**) cohort C. Vertical axis indicates number of reads corresponding to percentage of GC content represent by horizontal axis. All cohorts exhibit normal distribution for GC content, peaks at the expected 40-50% range.

	cuffdiff2	deseq2	EBSeq	edgeR	voom	voom+weight
**Cohort A**						
cuffdiff2	35	1	0	1	3	6
deseq2		3	0	1	0	0
EBSeq			1	0	0	0
edgeR				2	2	2
voom					8	7
voom+weight						17
**Cohort B**						
cuffdiff2	26	0	6	9	1	1
deseq2		6	0	4	1	1
EBSeq			11	8	0	0
edgeR				31	2	2
voom					11	8
voom+weight						11
**Cohort C**						
cuffdiff2	63	0	0	1	5	3
deseq2		3	0	0	0	0
EBSeq			71	0	0	0
edgeR				4	1	1
voom					19	10
voom+weight						14

Supplementary Table 1. Number of differentially expressed genes for the comparison of ADT and CRM. Number of common differentially expressed genes between pair method is represented by method from row and column.

DEG tool	Normalization method	Expression calculation	Alignment tool
cuffdiff2	geometric	FPKM	TopHat
deseq2	TMM	Htseq-count	TopHat
EBSeq	Median	RSEM	STAR
edgeR	TMM	Htseq-count	TopHat
voom	TMM	Htseq-count	TopHat
voom+weight	TMM	Htseq-count	TopHat

TMM: trimmed mean of M-values, FPKM: fragment per kilobase read length per million mapped read

Supplementary Table 2. Description of differentially expressed genes analysis methods for ADT versus CRM

Gender	Age (Years)	Medications
**ADU_A**		
F	4	NA
F	3	NA
F	16	NA
F	11	NA
F	7	NA
F	9	NA
F	7	NA
F	8	NA
**ADT_A**		
F	9	MTX
F	4	MTX
F	3	MTX
F	7	MTX
F	7	MTX
F	12	MTX, infliximab
F	5	MTX
F	5	MTX
**CRM_A**		
M	10	MTX
F	12	MTX
F	11	MTX
F	14	MTX, etanercept
F	10	MTX, etanercept
F	10	MTX, etanercept
F	8	MTX, etanercept
F	9	MTX
**HC_A**		
F	14	NA
F	9	NA
F	7	NA
F	11	NA
F	7	NA
F	10	NA
F	12	NA
F	13	NA

Supplementary Table 3a. Cohort A sample information. MTX: methotrexate, NA: not applicable/available

Gender	Age (Years)	Medications		Interval between sample
**ADT_B**		**ADT_B1**	**ADT_B2**	
F	7	MTX, etanercept	MTX, etanercept	4 months
F	5	MTX, infliximab	MTX, infliximab	4 months
F	11	MTX, etanercept	MTX, etanercept	5 months
F	8	MTX, etanercept	MTX, etanercept	4 months
F	5	MTX	MTX	2.5 months
F	12	MTX	MTX	5 weeks
M	3	MTX	MTX	3 months
F	4	MTX	MTX	6 weeks
**CRM_B**		**CRM_B1**	**CRM_B2**	
F	15	MTX	MTX	3 months
F	9	Etanercept	Etanercept	5 months
M	9	Etanercept	Etanercept	5 months
M	13	MTX, etanercept	MTX, etanercept	5 months
F	11	MTX, naproxen	MTX, naproxen	4 months
F	10	MTX, etanercept	MTX, etanercept	4 months
M	15	MTX, etanercept	MTX, etanercept	4 months
F	7	Etanercept	Etanercept	7 months
M	8	MTX, etanercept	MTX, etanercept	6 months
F	16	MTX, etanercept	MTX, etanercept	4 months

Supplementary Table 3b. Cohort B sample information. MTX: methotrexate

Gender	Age (Years)	Medications
**ADU_C**		
F	16	NA
F	11	NA
F	10	NA
F	6	NA
F	2	NA
F	15	NA
F	7	NA
F	16	NA
**ADT_C**		
F	12	NA
M	12	NA
F	7	NA
F	6	NA
M	12	NA
F	7	NA
NA	NA	NA
F	7	NA
**CRM_C**		
M	14	Methotrexate
F	9	Methotrexate
F	18	NA
F	15	Methotrexate
NA	NA	IL1 inhibitor
M	16	Etanercept
M	13	Methotrexate
F	14	Methotrexate
**HC_C**		
F	13	NA
F	8	NA
F	16	NA
F	4	NA
F	9	NA
F	15	NA
F	16	NA
F	14	NA

Supplementary Table 3c. Cohort C sample information. MTX: methotrexate, NA: not applicable/available

Method	FC	APV
**ADT_B1 vs ADT_B2**		
cuffdiff2	2	0.05
deseq2	1.2	0.10*
EBSeq	2	0.10
edgeR	2	0.05
voom	2	0.05*
voom+weight	2	0.05*
**CRM_B1 vs CRM_B2**		
cuffdiff2	2	0.05
deseq2	2	0.001*
EBSeq	2	0.05*
edgeR	2	0.001*
voom	2	0.05*
voom+weight	2	0.01*

FC: fold change, APV: adjusted p-value, *: p-value as cutoff

Supplementary Table 4a. Setting for differentially expressed genes identification by different methods for the comparison of two time points cohort B samples.

	cuffdiff2	deseq2	EBSeq	edgeR	voom	voom+weight
**ADT_B1 vs ADT_B2**						
cuffdiff2	19	0	0	2	2	0
deseq2		12	0	0	1	0
EBSeq			1	0	0	0
edgeR				11	0	0
voom					2	0
voom+weight						6
**CRM_B1 vs CRM_B2**	17	0	0	3	1	0
cuffdiff2		3	0	2	1	1
deseq2			0	0	0	0
EBSeq				13	2	1
edgeR					2	1
voom						1
voom+weight						

Supplementary Table 4b. Number of differentially expressed genes for the comparison over two time points for samples from cohort B ADT and CRM group. Number of common differentially expressed genes between pair method is represented by method from row and column.

	Cohort A		Cohort B		Cohort C	
	Mapping rate	Mapped reads	Mapping rate	Mapped reads	Mapping rate	Mapped reads
Average	86.58	41,593,196	90.49	35,464,982	83.78	50,828,966
Maximum	90.10	52,056,978	92.90	59,751,220	85.50	60,591,196
Minimum	78.00	22,557,546	75.10	10,588,944	78.80	40,001,700

Supplementary Table 5. RNA sequencing reads mapping to UCSC hg19 human reference genome

Number of raw reads	Cohort A	Cohort B	Cohort C
Number of sample, n	32	19 pairs	32
Average	62,262,707	52,106,104	74,263,403
Maximum	76,363,956	88,661,806	87,736,850
Minimum	50,392,008	15,608,508	60,709,052
Range	25,971,948	73,053,298	27,027,798
Coefficient Of Variance, COV (%)	10.059	28.693	9.916

COV= Average/Standard deviation*100%

Supplementary Table 6. Pre-alignment sequencing reads

	Exons	Intron	TSS up 10k	TES down 10k
**Cohort A**				
Average	77.608	18.038	1.289	3.065
Maximum	82.923	25.047	1.560	3.528
Minimum	70.564	12.701	0.966	2.489
**Cohort B**				
Average	77.212	19.219	0.976	2.594
Maximum	79.711	23.383	1.101	2.829
Minimum	72.892	16.841	0.873	2.314
**Cohort C**				
Average	78.249	17.246	1.240	3.265
Maximum	81.049	21.665	2.238	4.720
Minimum	74.160	14.506	1.046	2.756

TSS: transcription start site, TES: transcription end site

Supplementary Table 7. Genomic distribution of sequencing reads in percentage
